# Trap colour strongly affects the ability of deep learning models to recognize insect species in images of sticky traps

**DOI:** 10.1002/ps.8464

**Published:** 2024-10-08

**Authors:** Song‐Quan Ong, Toke Thomas Høye

**Affiliations:** ^1^ Department of Ecoscience Aarhus University Aarhus Denmark; ^2^ Institute for Tropical Biology and Conservation Universiti Malaysia Sabah Kota Kinabalu Malaysia; ^3^ Arctic Research Centre Aarhus University Aarhus Denmark

**Keywords:** glue trap, transfer learning, deep convolutional neural network, RGB sensor, artificial intelligence, precision biodiversity

## Abstract

**BACKGROUND:**

The use of computer vision and deep learning models to automatically classify insect species on sticky traps has proven to be a cost‐ and time‐efficient approach to pest monitoring. As different species are attracted to different colours, the variety of sticky trap colours poses a challenge to the performance of the models. However, the effectiveness of deep learning in classifying pests on different coloured sticky traps has not yet been sufficiently explored. In this study, we aim to investigate the influence of sticky trap colour and imaging devices on the performance of deep learning models in classifying pests on sticky traps.

**RESULTS:**

Our results show that using the MobileNetV2 architecture with transparent sticky traps as training data, the model predicted the pest species on transparent sticky traps with an accuracy of at least 0.95 and on other sticky trap colours with at least 0.85 of the F1 score. Using a generalised linear model (GLM) and a Boruta feature selection algorithm, we also showed that the colour and architecture of the sticky traps significantly influenced the performance of the model.

**CONCLUSION:**

Our results support the development of an automatic classification of pests on a sticky trap, which should focus on colour and deep learning architecture to achieve good results. Future studies could aim to incorporate the trap system into pest monitoring, providing more accurate and cost‐effective results in a pest management programme. © 2024 The Author(s). *Pest Management Science* published by John Wiley & Sons Ltd on behalf of Society of Chemical Industry.

## Graphical abstract

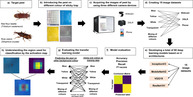

## 1 INTRODUCTION

Pest monitoring is an important element of integrated pest management (IPM) to enable sustainable management strategies based on accurate information on pest identification and numbers.^1^ One of the most common monitoring tools is the use of sticky traps with adhesive materials that can passively capture the target pests.^2^ Pests are not equally attracted to all colours of sticky traps and different coloured sticky traps are typically used for different pests. For example, green and yellow sticky traps are effective for catching aphids in pomegranates,^3^ while yellow sticky traps are commonly used for catching cranberry weevil.^4^ Pobozniak *et al*.^5^ compared the effectiveness of blue, yellow, and white sticky traps in monitoring thrips (Thysanoptera) in pea crops and found that the blue colour was the most effective in providing early warning to the crops. Doud and Phillips^6^ compared the pitfall trap and the transparent sticky trap in monitoring storage pests such as the red flour beetle (*Typhaea castaneum*), the hairy fungus beetle (*Typhaea stercorea*) and the grain weevil (*Ahasverus advena*) and found that the sticky trap caught significantly more fungus beetles and grain beetles than red flour beetles. Manual observation and counting of pests in the sticky trap is still the most common method, even if it is also very time‐consuming, labour‐intensive and prone to human error after long hours of work.

Traditional methods of pest detection include manual inspection, counting and taxonomic classification, which have long been used in the detection and control of pests. Usually, pest detection requires regular inspection by experienced personnel. For example, Doud and Phillips^6^ found that personnel monitoring pests in stored products had to check traps daily to weekly for about 2 months before and after performing control measures. This work did not include identifying the pest, which requires a further investment of time and labour. Therefore, image‐based approaches for detecting, counting, and classifying pests in sticky traps could be an alternative to reduce the labour and time required for manual identification and counting of pests. For example, Li *et al*.^7^ developed a deep learning model called TPest‐RCNN to detect whitefly and thrips using yellow sticky trap images from a glasshouse and achieved a mean F1 score of 0.94 and an average precision of 0.95. Böckmann *et al*.^8^ implemented the Bag of Visual Words (BoVW) algorithm in an automated system to distinguish between natural enemies and the species *Trialeurodes vaporariorum* and *Bemisia tabaci* and achieved a recall and precision of 0.85. Li *et al*.^9^ used You Only Look Once (YOLO) object recognition and support vector machines (SVM)‐based fine counting using global features to classify flying insects such as bees, mosquitoes and moths and achieved 0.93 accuracy in counting and 0.90 in classification. In most previous studies, the algorithm was trained based on a specific colour of the sticky trap, for example, yellow, or blue. However, this raises the question of generalisation of the model when applied to a different colour of sticky trap. In other words: How does the colour of the sticky trap affect the performance of the model? This is crucial because the deep learning algorithms used in automatic pest classification systems have difficulty distinguishing and classifying pests when confronted with a variety of background colours as they occur in the real world.^8^, ^9^

To overcome this challenge, transfer learning – a technique that uses a trained model's knowledge of a task^10^ to perform a new prediction task – could be an excellent solution.^11^ Transfer learning is a deep learning technique in which a data‐driven model previously trained for a specific task (e.g., pests on a yellow sticky trap) is retrained to create a new model for a related task (e.g., pests on a range of different colours), with less data needed for the new level of training. This technique can improve adaptability and robustness in dealing with the different backgrounds encountered in pest monitoring. For example, Pattnaik *et al*.^11^ demonstrated a transfer learning framework using the pre‐trained DenseNet169 that learned from ImageNet and achieved 0.89% accuracy in classifying pests in tomato plants. Ong *et al*.^12^ used a pre‐trained deep learning model – MobileNetV2 – and transferred the knowledge about the weighting of ImageNet to the prediction of mosquitoes collected in the field. They achieved a precision of 97% for internal test set, but later achieved an accuracy of 76% when applied in the field, where they hypothesised that the different backgrounds of the mosquitoes affected the performance of the model. Transferring knowledge from ImageNet,^13^ which includes 1000 object classes, is the most commonly used transfer learning framework for pest detection.^14^, ^15^, ^16^ In this study, we aim to use transfer learning in which deep learning was developed based on the knowledge learned from the training data of two common store product pest – red flour beetle (*Tribolium castaneum* H.) and rice weevil (*Sitophilus oryzae* L.) – on a single or mixed coloured sticky trap and later used to predict the pest on another coloured sticky trap. This study aims to make the following contributions:

1. Datasets showing pests on different coloured sticky traps.

To assess the adaptability and transferability of learning weight from one coloured sticky trap to another, we created datasets containing sticky traps with different background colours, including blue, yellow, white, transparent, and mixed four‐coloured traps. We also used three different recording devices, namely DSLR cameras, webcams, and smartphones, to capture the images of the sticky traps. The selection of colour is based on evidence that both beetles exhibit positive phototaxis. The rice weevil is more attractive to blue and green wavelengths, and red flour beetle is more attractive to near‐ultraviolet (UV) spectrum.

2 Deep learning models that can detect and predict pest identification in a range of different coloured sticky traps.

We have developed deep learning models with four algorithms: InceptionV3, MobileNetV2, VGG19 and ResNet101, to detect pests on four different coloured sticky traps. The selection of these four algorithms was based on the performance ranking at ImageNet^13^ and their performance in previous studies.^11^, ^12^, ^13^ The best models were used to evaluate the effectiveness of transfer learning. Here, the weights and biases learned from the best colour are used to predict the pest on other coloured sticky traps.

3 Visualisation of the activation region used to distinguish pests on different coloured sticky traps.

We used Grad‐CAM activation maps, which can visualise the deep learning models' attention and sensitivity to different images of pests on different coloured sticky traps and provide valuable insights into the influence of background colours on pest classification accuracy.

## 2 MATERIALS AND METHODS

**Figure 1:**
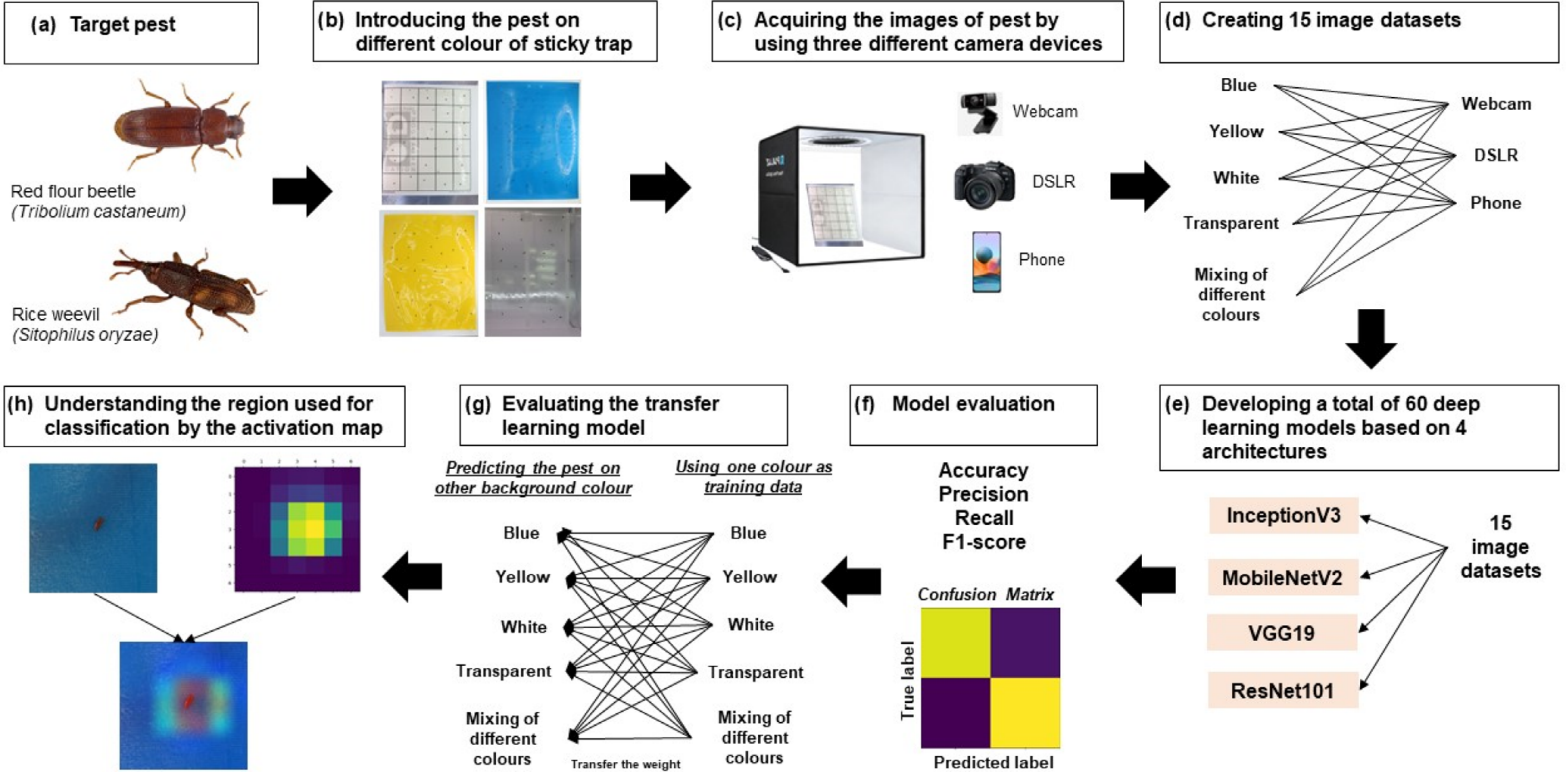
Overall methodology of the study on the influence of sticky trap colour on the performance of deep learning models in the detection of stored product pests.

The methodology of the present study is shown in Fig. 1. Briefly, the target pests were artificially placed on four coloured sticky traps and the images were captured using three different camera devices to generate image datasets that were used to develop deep learning models. The deep learning models were evaluated using matrices for accuracy, precision, recall and F1‐score. The background colour that gave the best results for the models was used as training data and other coloured sticky traps were predicted to evaluate the performance of transfer learning. Finally, the activation map was used to understand the region used by the neural network to discriminate pests on different coloured sticky traps.

### 2.1 Source of the pest and the sticky trap

We simulated the pest on the sticky trap using two common store product pests: the red flour beetle (*Tribolium castaneum*) and the rice weevil (*Sitophilus oryzae*), which were bred in the insect laboratory of the Institution for Tropical Biology and Conservation (ITBC), Universiti Malaysia Sabah, Kota Kinabalu, Malaysia. Both insect colonies were kept at a temperature of 25 ± 2 °C and 70 ± 5% humidity. The 5–6‐week‐old adult beetles were used for the experiment. The beetles were separated from the rearing media and killed with 95% ethanol. The insects were added to the sticky trap with tweezers. Each sticky trap was loaded with 15 red flour beetles or rice weevils and five replicates of each colour were prepared. All sticky traps were two‐sided sticky traps measuring 25 cm × 20 cm (CityFarm®, Malaysia).

### 2.2 Image acquisition

To examine the role of the sticky trap colour and camera system on image recognition performance, we designed an experiment where individuals of the two insect species were placed on four different colours of sticky traps and imaged with three different camera systems. We generated pest image data on yellow, blue, white, transparent sticky traps by using a digital single lens reflex (DSLR) camera (Canon EOS RP), a web camera (Logitech C922 Pro) and a mobile phone (Xiaomi Redmi note 9 Pro) with the technical details provided in Table 1. The images were captured according to the guidance provided by Ong *et al*.^17^ Briefly described, images were taken in a 30 cm × 30 cm × 30 cm photo studio box with white light illumination (PULUZ, Shenzhen, China). The equipment was stabilised with a tripod that was turned upside down and pointed at the box from above. The images were taken individually and saved in JPEG format. The image files were labelled and sorted according to their background colour and the equipment used to take the images. We created the datasets by capturing pest images of about 300 specimens of the red flour beetle and the rice weevil, respectively, using three different devices – DSLR, webcam and mobile phone. Therefore, a total of 1800 images were acquired (300 per species × two species × three devices) for this study.

**Table 1:** Technical specification of the devices and settings used to take the images

Type of imaging device	DSLR and mirrorless camera system	Mobile phone	Webcam
Brand and model	Canon EOS RP	Xiaomi Redmi note 9 Pro	Logitech C922 Pro
Camera sensor spec	26.2MP Full‐Frame CMOS Sensor	64 MP, f/1.9, 26 mm (wide), 1/1.72″, 0.8 μm, PDAF	2 MP, CMOS 1/2.7
Lens	Tamron SP AF 90 mm f/2.8 Di Macro	‐	‐
Technical setting	ISO 800 auto white balance	ISO 800 auto white balance	Full 1080p at 30fps auto white balance

### 2.3 Pre‐processing and augmentation of data

The size of the dataset was planned on the basis of previous studies^12^, ^13^, ^17^, ^18^ in which a total of 1800 images were acquired. However, this was limited by the number of pests available on the sticky trap. The data was split into separate subsets for training (70%), testing (15%) and prediction (15%). Augmentation was performed after splitting the data to avoid using the same original data only for either training, testing or prediction. Before developing the deep learning models, a series of data augmentation techniques were performed where all images were rotated by 0°, 90°, 180° and 270° so that the total number of images eventually totalled 7200 (4 × 1800 images). The images were normalised to obtain a range of [0, 1] pixel values and a resolution of 224 × 224.

### 2.4 Development of deep learning models and transfer learning to other coloured sticky traps

We unfroze the convolutional blocks of the pre‐trained convolutional neural networks (CNNs) and re‐trained most of the parameters as described in Ong *et al*.^18^ for four deep learning architectures – InceptionV3, MobileNetV2, VGG19 and ResNet101. We evaluated the effectiveness of transfer learning by using knowledge learned from high‐performing architectures to predict pests on sticky traps with different background colours. To achieve this, each of the colours was used as training data and the pest with others background colours were predicted, for example, using blue colour data to predict the pest on yellow, white, and transparent, respectively. The selection of these hyperparameters was based on Ong *et al*.,^18^ where the deep learning models were trained using the adaptive learning rate optimisation algorithm (ADAM). The performance of these models was evaluated using four metrics: accuracy, precision, recall and F1‐score. We used the Keras deep learning framework on an NVIDIA Tesla P100 PCIE GPU platform to train and evaluate the models. The training was performed for 100 epochs, and the learning rate was 0.00001 with 32 batches.

The differences between the model performances were first determined by a two‐way factorial analysis of variance (two‐way ANOVA; SPSS 22.0) at a significance level of *α* = 0.05, using both background and device type as independent variables and the F1 score (the harmonic mean of accuracy and precision), which represents the model performance, as the dependent variable. For transfer learning, significant differences in these dependent factors – the F1 score – were compared using the 95% confidence interval (CI) of the standard error (SE) of the F1 score, with no overlap indicating rejection of the null hypothesis.^19^ In addition, a generalised linear model (GLM) was performed to determine the strength of prediction of three variables – sticky trap colour, devices, and deep learning architecture – on model performance (F1 score). To further rank the importance of the variables, we use an additional selection algorithm called Boruta,^20^ a random forest‐based wrapper algorithm that filtered relevant variables by comparing the mean of the original variables with the random achievable mean estimated from their permuted copies.

### 2.5 Activation map to distinguish pests on different coloured sticky traps

To gain further insight into the influence of the background colour of the pest, we created activation maps to visualise the model's attention and sensitivity to different colours. These maps provide valuable information about the influence of background colour on the classification process. We used Gradient‐weighted Class Activation Mapping (Grad‐CAM) to visualise the range used by the neural network to classify the pest with a variety of coloured backgrounds. In general, we retrieved one layer at a time to extract low‐level and high‐level features from the architecture that explain why the colour performs better compared to other colours.

## 3 RESULT

### 3.1 Pest on sticky trap dataset

**Figure 2:**
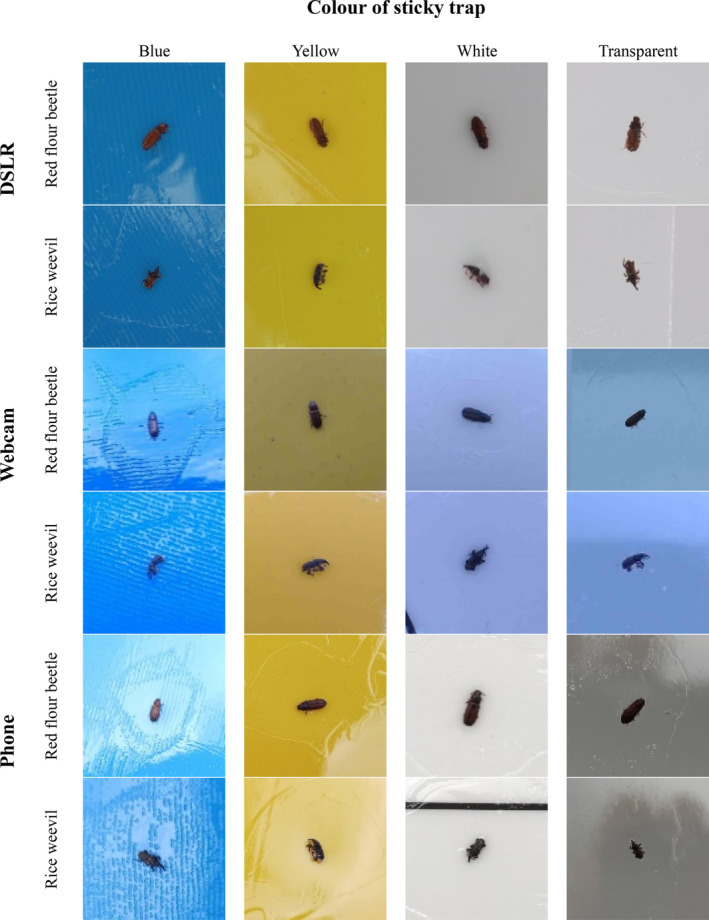
Examples of images of red flour beetles and rice weevil on four coloured sticky traps taken with three different devices.

Figure 2 shows the result of the dataset, which consisted of labelled images captured by three devices with sticky traps of four different colours. To understand the generalisation of the model in learning a mixture of background colours, we constructed an additional dataset for each imaging device contained a mixture of four colours. This dataset consisted of a mix of images from the other four datasets. This resulted in a total of 15 datasets (four background colours and a mixture of four colours when using three devices). The datasets are publicly available on Figshare at https://doi.org/10.6084/m9.figshare.23617383.v2.^21^ As can be seen from the clarity of the pests in the images in Fig. 2, the variation in the RGB (red, green, blue) sensors used in this study has an impact on the output quality of the images.

### 3.2 Development of deep learning models and transfer learning performance

Before implementing the model on a variety of coloured sticky traps (to transfer the knowledge of identifying pests with different background colours), we identified the architectures and colours that have high performance in predicting the single‐colour sticky traps (same background colour for training, testing and prediction data). In general, MobileNetV2 with a transparent background performed best with an accuracy of 0.95 and an F1 score of 0.95, while the architecture InceptionV3 showed the most balanced and stable performance across all colours (except blue) and imaging devices. Regarding the colour effect of the sticky trap, the deep learning model performed quite well in detecting the pest on the blue sticky trap across all architectures and devices, especially on the images captured with the webcam and ResNet architecture. The Appendix 1 shows the details of model performance result for each model as a function of architecture, imaging devices and sticky trap colour.

**Figure 3:**
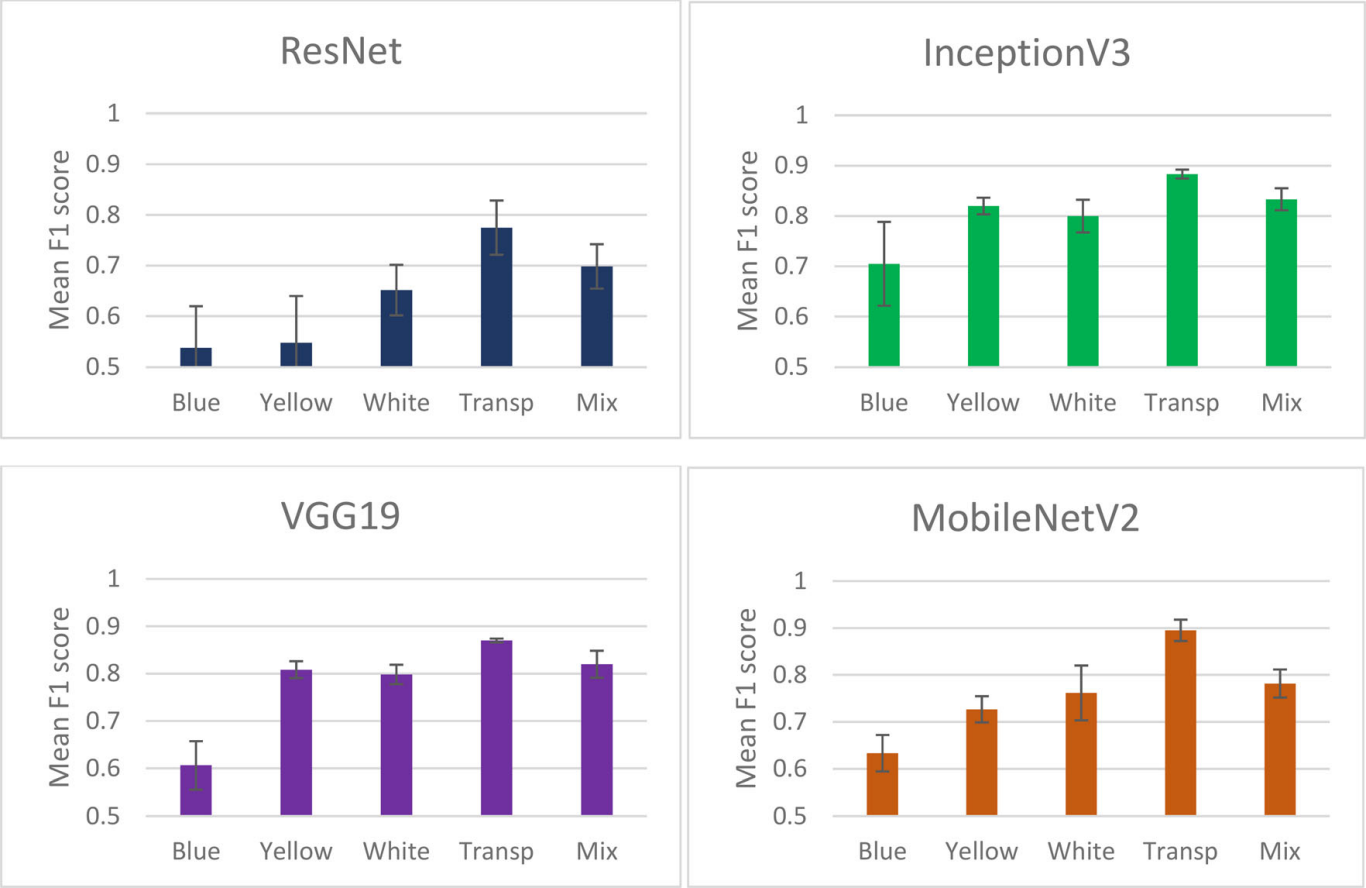
Mean F1 score with standard error (SE) bars for the performance of the deep learning models in classifying pests using five different datasets – yellow, blue, white, transparent and a mixture of different colours.

**Figure 4:**
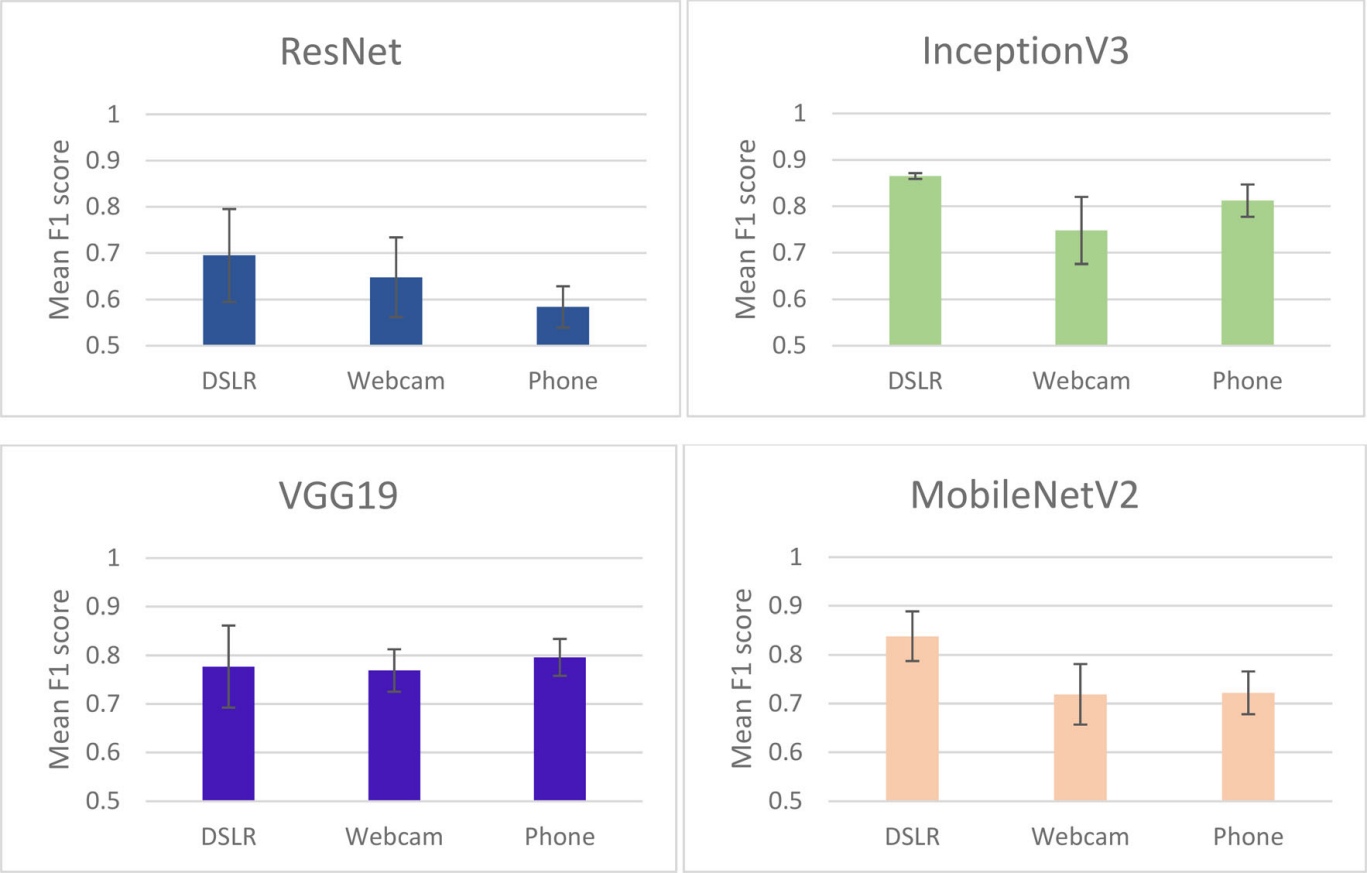
Mean F1 score with standard error (SE) bars for the performance of the deep learning models in classifying pests based on three devices – DSLR, webcam and phone.

**Figure 5:**
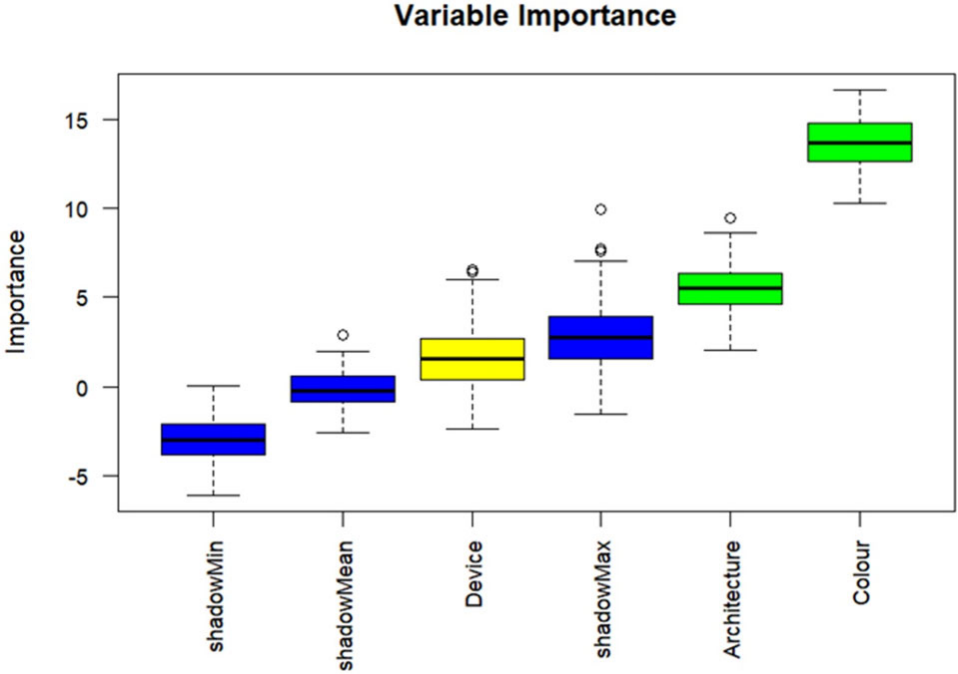
Variable importance ranking through BORUTA algorithm: the green boxplots indicate the confirmed important variables; the yellow boxplots indicate the uncertain features. Blue boxplots serve as reference levels corresponding to the maximum, average, and minimum *Z* scores of a shadow variable.

The result of the two‐way ANOVA shows that both colour and devices have a significant impact on the performance of the deep learning model when using the MobileNetV2 (*F*_2,4_ = 65.71, *P* = 0.035) and InceptionV3 (*F*_2,4_ = 157.08, *P* = 0.021) architectures. This is especially true for the model trained and tested with a transparent background (Figs 3 and 4). This suggests that the architectures trained with the transparent background of the sticky trap are potentially transferable and can predict the pest on other background colours. Furthermore, our results for the ResNet and VGG19 architectures using three different devices to detect pests on different coloured sticky traps show no significant differences between the devices. The result of the GLM showed that the F1 score was significantly associated with the variables colour, devices and deep learning architectures (Table 2, *F*_3,56_ = 15.37, *P* < 0.001, Adjusted R‐squared = 0.42). To understand which variables might have a greater impact on the model's performance (the F1 score), a random forest‐based wrapper algorithm (Boruta) was used to rank the variables. Boruta ranked both sticky trap colour and the deep learning architecture as significantly more important than the devices in predicting the F1 score (Fig. 5), as these two variables significantly predicted the F1 score. This suggests that changing the background colour and deep learning architecture would significantly affect the performance of the deep learning model.

**Table 2:** The result of the generalised linear model (GLM) with the predictor of the colour of the sticky traps, the devices and the deep learning architecture on the F1 score

Variables	Estimate	Standard error	*t*‐Value	*P*‐Value
Intercept	0.568	0.053	10.816	<0.0001
Sticky trap colour	0.046	0.009	4.976	<0.0001
Devices	−0.036	0.016	−2.297	0.025
Deep learning architecture	0.046	0.012	4.009	0.00018

*Note*: Residual standard error: 0.10 on 56 degrees of freedom (DF); Multiple R‐squared = 0.45; Adjusted R‐squared = 0.42; F‐statistic: 15.37 on 3 and 56 DF, *P*‐value < 0.0001.

**Figure 6:**
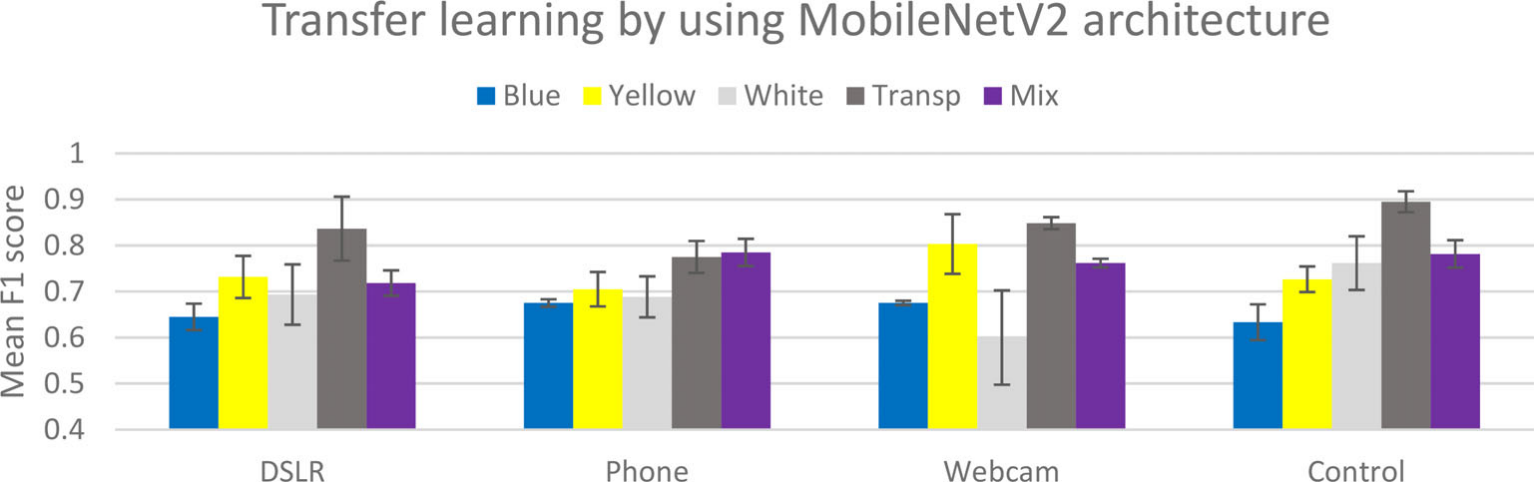
Using MobileNetV2 architecture with images of the transparent sticky traps as the training data to predict other coloured sticky traps.

From the result in Fig. 3, the model developed with two architectures – InceptionV3 and MobileNetV2 – generally performs best, and therefore was used for the transfer learning study. Each of the colours was used as the training data and later used to predict the pest on the other colours that were acquired by the three devices. For example, we used images acquired by DSLR (independent variable in the *x*‐axis of Fig. 6) and predict pest on different background acquired by the three devices (DSLR, phone and Webcam). Mean and SE of F1‐score for the prediction validate the model statistical significance by the overlapping SE bars. We also benchmark the transfer learning by using a control, where the training and prediction data have the same colour. We found that models trained on images of the transparent sticky traps for the MobileNetV2 architecture performed significantly greater than models trained on images of traps of the other colours, for example, by using yellow and blue colour as the training data.

### 3.3 Visualisation of the regions used by the architecture for classification

In order to understand why the model with transparent sticky traps performs better than other coloured sticky traps, we created the activation heat map with Grad‐CAM. This algorithm allows us to visualise the regions extracted as features for classification. The important regions were shown in the yellow‐orange‐red colour spectrum. We covered the low‐level features by the inverted residual bottleneck block 4, where the semantic segmentation of feature extractors focuses on shape and line of the object.^22^ Table 3 shows that the activation map mainly distinguishes between object and background. From the observation of the map, the transparent sticky trap with the MobileNetV2 architecture provide a clear separation of object and background compared to the blue and yellow ones.

**Table 3:** Visualisation of the low‐level features region using the inverted residual bottleneck block 4 of the MobileNetV2 architecture

		Blue	Yellow	Transparent	White
Red flour beetle	Input image	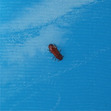	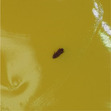	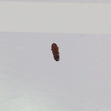	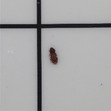
	Activation	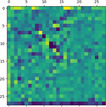	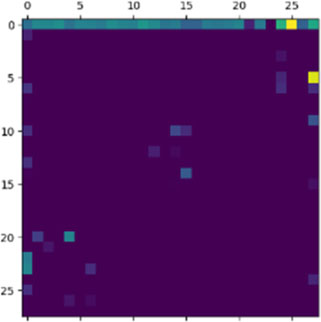	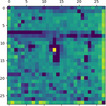	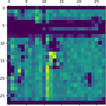
	Overlapped	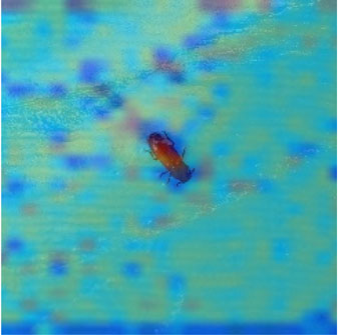	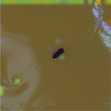	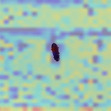	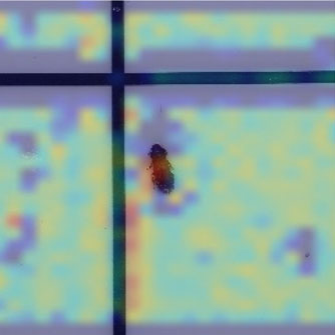
Rice weevil	Original	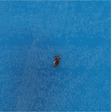	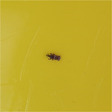	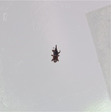	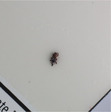
	Activation	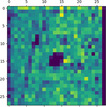	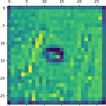	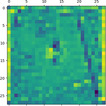	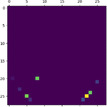
	Overlapped	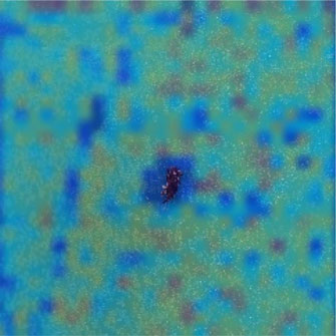	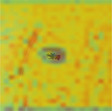	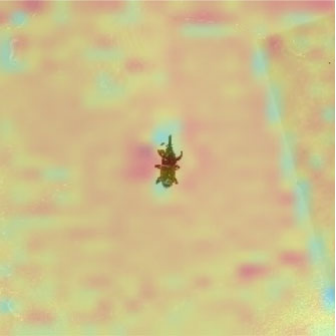	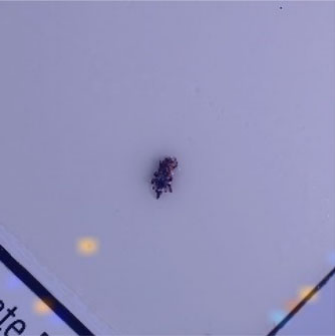

Since the morphological features of the red flour beetle and the rice beetle are different, we cover the block of high‐level features in the MobileNetV2 architecture. The linear projection layer is a high‐level feature extraction block that reduces the number of channels, serves as a bottleneck and facilitates high‐level feature extraction. We visualised block 16 of the linear projection layer and as shown in Table 4, the yellow area of both the red flour beetle and the rice beetle on the transparent sticky trap showed higher coverage than other colours, for example the blue sticky trap. This could be the main reason why the transparent sticky trap allows the architecture to extract more features from the pest that can be learned for classification.

**Table 4:** Visualisation of the high‐level features region using the linear projection layer block 16 of the MobileNetV2 architecture

		Blue	Yellow	Transparent	White
Red flour beetle	Original	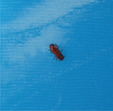	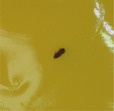	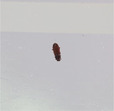	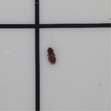
	Activation	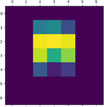	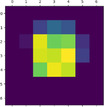	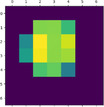	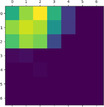
	Overlapped	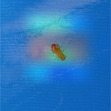	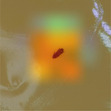	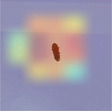	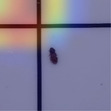
Rice weevil	Original	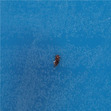	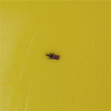	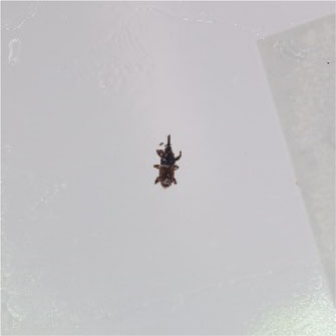	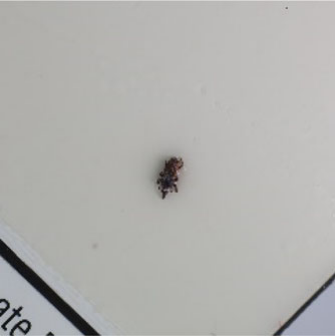
	Activation	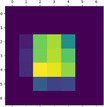	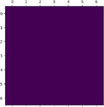	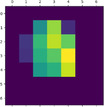	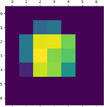
	Overlapped	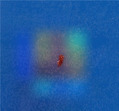	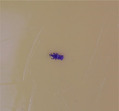	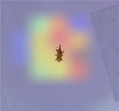	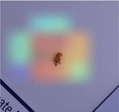

## 4 DISCUSSION

In an integrated pest management programme, the use of sticky traps to monitor pests is crucial to detect pest species and determine their abundance. To make pest monitoring cost effective, we implemented a computer vision and deep learning model to detect and classify the pests of two store products based on the different colours of the sticky traps. Our dataset contained more images of pests on coloured sticky traps compared to previous studies using only the yellow sticky trap^7^, ^8^, ^23^, ^24^, ^25^, ^26^, ^27^, ^28^ and we achieved at least 0.98 accuracy on the test set. Importantly, we found substantial variation in model performance among colours of sticky traps. Even though, the yellow sticky trap is the most commonly used sticky trap, several different colours are used in insect pest monitoring.^2^ The problem is that if the training data consists only of yellow stricky traps, a deep learning model may perform more poorly when confronted with images of different coloured sticky traps.

Our results show that using a transparent background for the pest results in the highest predictive performance of our deep learning models. To our knowledge, pest detection on transparent sticky traps has not yet been studied in terms of computer vision and deep learning model performance. There could be two main reasons for why transparent sticky traps are rarely used. The first is the effectiveness of the transparent sticky trap in catching the pest. The effectiveness of a transparent sticky trap in attracting pests has been investigated in several studies. For example, Dimitrova *et al*.^29^ compared yellow and transparent sticky traps in an agricultural ecosystem with olive trees and concluded that the transparent sticky trap did not catch significantly more insects compared to the yellow sticky trap. Kardinan and Maris^30^ studied the attractiveness of different coloured sticky traps in a garden with red chillies and found that white and yellow sticky traps performed better than transparent sticky traps for *Bactrocera* sp. and general insects. The second reason is the technical problems with image capture. These technical problems include the difficulty of focusing on the pests (especially with mobile phones and webcams that activate autofocus mode) because the contrast between the object and the clear background colour is too low.

Our study addresses one of the most common problems in the acquisition of image data for the development of automatic classification, namely the image acquisition equipment and the quality of the images. The result shows that there is no significant difference in the performance of deep learning models when web cameras and smartphones are used as image capture devices. This suggests that less expensive and widely available devices can be used effectively for pest monitoring, making the implementation of such systems accessible to a wider audience. However, one of the most important findings from the comparison of the devices in model generalisation is that when the DSLR was used as training data, the models generalised poorly for the images captured with the webcam and the phone (Figs 5 and 6). This shows the importance of not only using high quality images as training data for the development of the deep learning. This result also highlights the importance of considering a variety of image qualities and recording devices when training the model in order to achieve better generalisation and robustness in real‐world applications. Morikawa *et al*.^31^ pointed out that the main difference between mobile devices and DSLRs is the size of the image sensors, with DSLRs having a much larger sensor and therefore being able to produce higher resolution images. Nevertheless, our result suggests that higher quality images generated by DSLR could poorly predicted the lower resolution or quality images that generated by mobile phone and web camera. Our result contrasts with the result of Thambawita *et al*.^32^ who found that the performance of the model increased with higher image resolution. Our result, which included a means of statistical validity, states that the resolution of the images is not significantly different, especially when the resolution was standardised to 224 × 224 pixels [the most common input resolution for deep convolutional neural network (DCNN)].

In this study, a total of 60 models (15 datasets × 4 deep learning architectures) were developed to compare the performance of the models in classifying pests on different coloured sticky traps. Our result generally supports the studies of Spiesman *et al*.^33^ that InceptionV3 performed best among ResNetm Wide ResNet and MnasNet models in classifying bumblebee species. Our result showed the high adaptability and consistency of performance of InceptionV3 in different coloured sticky traps. This result is in line with the findings of Ong and Hamid,^34^ who compared four deep learning architectures in classifying insects according to their taxonomic level, where InceptionV3 showed the most consistent performance across different levels. In terms of transfer learning, most previous studies have used the architecture as a pre‐trained model by training the architectures' extraction layers with features learned from ImageNet and transferred the weights and biases to other classification tasks.^13^ In contrast, this study aims to use the learning ability of the architecture for the pests on different coloured sticky traps, and therefore we trained the entire architecture – from feature extraction to classification layers. A similar approach has been used in previous studies,^18^ which started with the feature extraction part with a set of convolutional layers that apply a set of filters to detect the presence of features for the input image data. Furthermore, our result from Boruta shows that the colour of the trap and the architecture of the model were significantly more important than the device, suggesting that more emphasis should be placed on the development of the architecture to improve the performance of the model.

**Figure 7:**
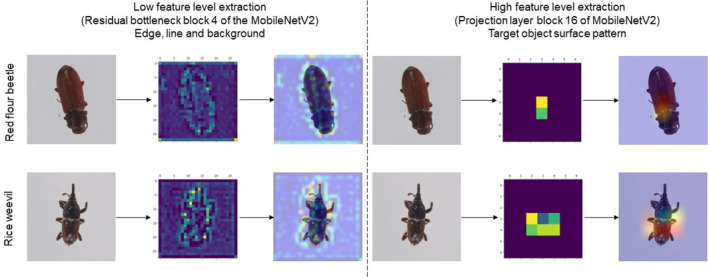
The low‐ and high‐level features visualised with the Grad‐CAM algorithm. Low‐level features extract the edges of the pests, while the high‐level features extract the surface pattern of the pest. All extracted features eventually contribute to the classification.

From a pest management perspective, red flour beetles (*Tribolium castaneum*) and rice weevils (*Sitophilus Oryzae*) cause significant quantitative and qualitative damage to agricultural products during postharvest storage. Identification of pests is crucial in the context of IPM, and both the red flour beetle and the rice weevil can be identified by their morphology. For example, the head and upper part of the thorax of the red flour beetle have tiny punctures and the elytra are longitudinally grooved^35^; the rice weevil has a long snout that accounts for almost one‐third of the total length and the elytra each have two pale orange spots.^36^ To understand the pest morphology (as the extracted features in machine learning) used by architecture to distinguish the red flour beetle and the rice beetle, Grad‐CAM is an excellent tool to obtain the activation map and visualise the region from the image. The activation maps, often referred to as feature maps, were a collection of feature activations detected by neurons in each convolutional layer and have dimensions of height × width × channels. These feature maps were successively processed as input by the next convolutional layer to recognise the higher‐level features. Since we used the algorithm Grad‐CAM to generate the heat map on the layers of the model, these features can be projected and visualised as a region for classification, which offers great possibilities for taxonomy of pests. In this way, the computer can help the human expert identify some regions that are difficult for the human eye to see but can be detected by the sensor. As can be seen in Fig. 7, the low‐level feature is able to extract the edges of the two pests, while the high‐level features are able to identify the surface pattern of the pest and eventually contribute as features to the classification. Since the morphological features of the beetles in this study have a different shape, we need to visualise several levels, not only the last convolutional level. For example, the 16th folding level heatmap located coarse‐grained areas of differentiation between image classes and clearly distinguished the shape of the specimen, while the fourth folding level heatmap in Fig. 7 highlighted the elytra of the two specimens that had distinguishable morphological features.

In the real world of pest control, deep learning models have been widely used in microcomputers (Raspberry Pi and Arduino) or edge devices, which have simple specification requirements and can be easily used to collect data in many situations.^37^, ^38^ Indeed, there are many applications in this field, especially in agriculture, which can be integrated with other sensors or the Internet of Things (IoT) to form an agricultural precision system.^39^ Nevertheless, there are still many challenges in practise, such as type of imaging system, power supply and internet connectivity, which are critical to the successful implementation of these technologies. This study supports the pest detection of the precision system, whereby the selection and design of the colour of the sticky traps and the deep learning architecture should precede the deployment of the system in the field. The cost of implementing the system could be relatively high initially.^40^ However, in the long term, the use of the system in a large pest monitoring area, for example crops and stored products, would be worthwhile for the farmer or owner.^38^, ^39^ As the costs also depend on the business model, a subscription model, for example, could significantly reduce the initial costs for the farmer. As shown in some examples,^39^, ^40^ the provider could receive the relevant pest data in the meantime to actively update the model's performance.

In addition, this study also recognises some limitations. Firstly, the transparent sticky trap provides a good result in deep learning detection, but transparent sticky traps are only used to control some pest species.^6^ Therefore, there are some strategies that could overcome this limitation, such as providing ‘colour’ in the background of the transparent sticky card to attract other insects. For example, thrips are more attracted to yellow colour,^4^, ^5^ and a yellow background can be placed on the transparent sticky card and eventually serve as a yellow sticky trap. The transparent sticky card can then be removed from the trap and used to analyse the deep learning model. A second limitation is that our training data does not include images taken in the field (*in situ*) with a wider exposure range, as our study design consists of bringing the sticky card to the laboratory to image, count and identify the insects. We decided on this approach to achieve optimal image quality while still proposing a cost‐effective method to process and monitor insect biodiversity from sticky cards. Future research focusing on real‐time *in situ* detection will require a wider variety of training data, especially under different illumination conditions found in the field. A third limitation is that this study only focuses on two pest species. The effectiveness of the models might be different if they were applied to a wider range of pests, especially those with different morphologies. However, as the aim of the studies focused on the effects of the colour of the sticky traps, the results are still relevant to answering the research question. In future studies, the pest dataset could be extended to other pests. However, this would depend on the research question to be investigated, especially the type of crop and region, or on a more general fundamental model that could be used to recognise and classify most insects.^41^ The fourth limitation is that the deep learning models developed and studied were used for classification tasks, while some of the state‐of‐the‐art algorithms are actually used for object detection and segmentation. Therefore, future research could investigate object detection models such as YOLO and Single‐Shot Detector (SSD) to evaluate their performance in accurately localising pests on sticky traps. The problem with these models is also that the background colour is different, which ultimately affects the performance of the model.

In summary, this study addresses the critical need for the development of an automatic pest identification system for sticky traps and assesses the importance of pest background colour, imaging device variation and deep learning architecture. The use of deep learning models with transfer learning can significantly improve the efficiency and accuracy of pest classification and enable timely and targeted intervention. The results of this research contribute to the advancement of automated pest monitoring systems and enable more accurate and timely pest control interventions in various areas, ultimately safeguarding public health, protecting valuable resources, and promoting environmental sustainability.

## AUTHOR CONTRIBUTIONS

Both authors conceived the study, carried out the analysis, wrote the draft, revised the manuscript critically, and approved it for publishing.

## CONFLICT OF INTEREST STATEMENT

Both authors declared no competing interests.

## Data Availability

The data that support the findings of this study are openly available in Annotated images of pests on different coloured sticky traps at https://doi.org/10.6084/m9.figshare.23617383.v2.

## Appendix 1

The result of the performance of each model based on the architecture, the devices, and the colour of the adhesive trap.
